# Adverse obstetric symptoms and rural–urban difference in cesarean delivery in Rupandehi district, Western Nepal: a cohort study

**DOI:** 10.1186/s12978-016-0128-x

**Published:** 2016-03-01

**Authors:** Vishnu Khanal, Rajendra Karkee, Andy H. Lee, Colin W Binns

**Affiliations:** Nepal Development Society, Bharatpur, Nepal; School of Public Health and Community Medicine, BP Koirala Institute of Health Sciences, Dharan, Nepal; School of Public Health, Curtin University, Perth, WA Australia

**Keywords:** Cesarean section, Maternal health, Nepal, Obstetric complications, Stillbirth

## Abstract

**Background:**

The burden of maternal morbidity is high in developing countries including Nepal. This study investigated obstetric complications and rural–urban difference in cesarean delivery rate in Western Nepal.

**Methods:**

A community-based cohort study was conducted in the Rupandehi district of Western Nepal during January-October, 2014, by interviewing 735 mothers within one month postpartum. The prevalence of obstetric complications was reported via frequency distribution, while factors associated with cesarean delivery were assessed using logistic regression analysis.

**Results:**

The prevalence of adverse obstetric symptoms during antenatal, intranatal and postnatal periods were 19.7 %, 27.8 % and 21.6 %, respectively. In total, 81 (11.0 %) mothers reported having stillbirths. The cesarean delivery rate was 14.1 % overall but was four times higher in the urban (23.0 %) than in the rural areas (5.8 %). Prolonged labor (19.0 %) and heavy bleeding (16.7 %) were common among rural women. Logistic regression analysis confirmed that cesarean section was more likely for mothers residing in urban areas than in rural areas (adjusted odds ratio 3.41; 95 % confidence interval 2.01 to 5.78).

**Conclusions:**

About one in five mothers reported some adverse obstetric symptoms. Obstetric problems were more common in the rural areas, whereas cesarean delivery rate was much higher in the urban areas. Further investigations are required to determine whether these cesarean sections are medically warranted or provider induced.

## Background

Worldwide, maternal mortality has fallen substantially at an annual rate of 1.3 % since 1990 [1]. In developing countries, the maternal mortality ratio was 283.2 per 100,000 live births in 1990 and decreased to 209.1 in 2013 [1]. Despite such progress, about 15 % of women suffer obstetric complications every year [2], while the burden of maternal morbidity remains high in developing countries, the consequences of which include infertility, psychological effects, disability, and loss of quality of life [3].

Nepal has achieved great success in reducing maternal mortality since 1990, notwithstanding severe political unrest, unstable economy and poor health infrastructure [4]. The maternal mortality ratio was 850 per 100,000 live births in 1990, reduced to 281 in 2005, and 170 by 2013 [5]. However, a recent survey in 2009 reported that 41 % of maternal deaths occurred in health facilities and 40 % at the home, suggesting that early notification and response to danger signs in these settings are essential to save the lives of Nepalese mothers [6].

With increasing health facility deliveries, the cesarean section rates in Nepal are also rising. There is some concern that the increase could be due to more planned or provider initiated operations rather than medically indicated [7]. In resource poor settings such as Nepal, provider-induced cesarean sections are likely to exert additional pressure on the already stretched health system. Therefore, monitoring of the cesarean delivery rate is important to ensure health equity particularly for disadvantaged populations and vulnerable groups, and that maternity services are being provided thoughtfully. It has been reported that 40 % of women experienced a serious illness during pregnancy and childbirth [8], while 21.1 %, 24.4 %, and 10.2 % of mothers had suffered from obstetric problems related to childbirth during the antenatal, intranatal and postnatal periods, respectively [7]. The present study aimed to determine the prevalence of obstetric complications and to investigate the rural–urban difference in cesarean delivery in Western Nepal. In view of the recent major earthquake which has destroyed infrastructure and many health facilities across the country [9, 10], our results are particularly timely and important to arouse worldwide attention to the problem.

## Methods

### Study setting

The Rupandehi district lies in south-western Nepal, with relatively good access to hospitals and other health facilities in the region. The district has three referral hospitals and one district hospital in urban areas, and five primary health care centers, six health posts and 58 sub-health posts in rural areas, providing services to its population of 880,196 (432,193 males, 448,003 females) [11, 12]. According to the 2009 Maternal Mortality and Morbidity Survey, the Rupandehi district has a higher maternal mortality (274 per 100,000 live births) than the national average (229 per 100,000 live births) [6]. The district is predominantly ‘rural’ consisting of village development committees, whereas ‘urban’ is defined as municipalities. While each village development committee has at least one government facility, health posts and sub-health posts do not have doctors and nurses stationed to provide full maternity services. Similarly, primary health care centers are not equipped to perform cesarean sections, unlike urban hospitals. Most rural areas are linked to the cities by gravel roads. Public transport is often irregular and non-functional during the rainy season due to road blockades, and therefore cannot be relied upon for medical emergencies. When public transport runs smoothly, it takes one-day travel to reach the nearby city from some of the rural areas for referral services.

### Participants and procedure

**Fig. 1:**
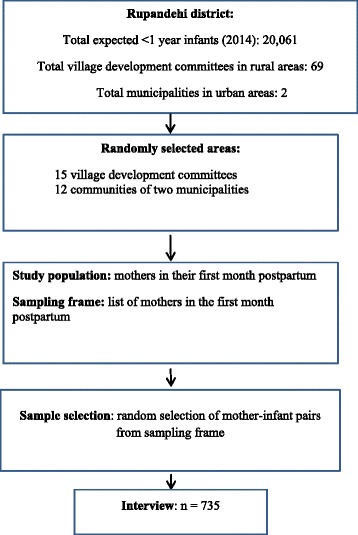
Study flow chart

A cohort study was conducted in the Rupandehi district during January-October, 2014. Fig. 1 explains the process of recruitment of participants [13]. Briefly, the total population of expected infants (<1 year) was 20,061 in 2014 [Source: District Public Health Office, Rupandehi, Annual Target 2013/2014]. A total of 735 mother-infant pairs (rural 378, urban 357) were recruited from 15 randomly selected village development committees and 12 communities of two municipalities. A few urban women (*n* = 18) declined to be interviewed whereas all women participated in the rural areas [14]. Lists of eligible mothers were compiled in the selected areas with the help of local female community volunteers. Recruitment from each community was proportionate to its number of expected infants per month. The inclusion criteria were: singleton birth; age of infant <30 days; resident of the community; and the child was alive at the time of recruitment.

Face-to-face interviews were conducted by trained female enumerators using questions adapted from the Nepal Demographic and Health Survey [15] and a previous questionnaire validated in central Nepal [7]. To obtain information on obstetric complications, common symptoms were read to mothers so that they could respond ‘yes’ or ‘no’ [16]. The list included: (1) antenatal: heavy bleeding (soaked clothes/bed/floor), swollen hands, fits or loss of consciousness, blurred vision, severe headache, severe fever, severe abdominal pain, and severe vomiting; (2) intranatal: heavy bleeding(soaked clothes/bed/floor), prolonged labor (12 hours or more), retained placenta (placenta not delivered within 30 minutes), swollen hands, and fits or loss of consciousness; (3) postnatal: heavy bleeding (soaked clothes/bed/floor), severe fever, foul smelling watery discharge, swollen hands, and fits or loss of consciousness. The choice was spontaneous and also kept as open ended to allow the recording of signs and symptoms not captured by our questionnaire. However, confirmation of obstetric morbidities through clinical audit was not feasible. Ethics approval of the study protocol was obtained from the Nepal Health Research Council (773/2014) and the Human Research Ethics Committee of Curtin University (approval number HR184/2013). Mothers provided consent for themselves. Personal identifiers were removed from the dataset before statistical analyses and only aggregated data were reported.

### Definitions and variables

The outcome variables were complications during pregnancy and childbirth. The method of delivery was either cesarean or vaginal. Stillbirth was specified as death of a fetus after 28 weeks of gestation [17] and recorded for the previous birth only. Prolonged labor was defined as lasting12 hours or more. Heavy bleeding was defined as bleeding that soaked clothes, bed or floor of the room [7].

Independent variables were identified based on literature review. We recoded maternal age (years) as: 15–19, 20–29, and 30–45; maternal education as: ‘no education’ , ‘primary/lower secondary’ (up to grade 8), ‘secondary’ (grade 9–10) and ‘higher’ (grade 11–12 and university degree). Paternal occupation was classified as: ‘employed’ (salaried job), ‘semi-employed’ (labor, business and foreign labor), and ‘unemployed’ (no paid job, and unpaid subsistent agriculture). Household assets (water source, toilet facility, types of cooking fuel, separate kitchen, floor material, electricity, radio, television, mobile phones and cupboard) were assigned scores using principal component analysis to generate a wealth index, which was then categorized into the lowest (poorest) to the highest (richest) quintile [16] and further re-grouped into three levels to facilitate analysis: ‘poor’ (lowest 40 %), ‘middle’ (middle 40 %) and ‘rich’ (upper 20 %) [18].

### Statistical analysis

The prevalence of obstetric complications, stillbirth and cesarean delivery were reported as the proportion of participants in this study. The rural–urban difference in cesarean section rate was confirmed by a multivariable logistic regression model with the backward stepwise method to control for confounding variables. All data analyses were performed using the Statistical Package for Social Sciences (IBM, SPSS version 20).

## Results

A total of 735 participants were enrolled in the study, whose characteristics are presented in Table 1. The average age was 24.6 (SD 4.6) years, with the majority (74.8 %) within the 20–25 years age group. About a quarter (26.0 %) of the women had no formal education. Most of their partners (70.0 %) were semi-employed, and one-fifth of participants (19.8 %) came from rich families. In terms of antenatal care, the great majority (76.2 %) of mothers paid four or more visits. In total, 314 (42.8 %) of them were primiparous and 88.2 % delivered in a health facility.

**Table 1:** Characteristics of participants, Nepal (*n* = 735)

Characteristics	Frequency	Percentage
Maternal age^a^		
15–19 years	99	9.0
20–29 years	549	74.8
30–45 years	119	16.2
Maternal education		
No education	192	26.0
Primary to lower secondary	243	33.1
Secondary and above	300	40.8
Paternal occupation		
Employed	102	13.9
Semi-employed	516	70.2
Unemployed	117	15.9
Place of residence		
Rural	378	51.4
Urban	357	48.6
Wealth status		
Poor	295	40.1
Middle	295	40.1
Rich	145	19.8
Maternal height^a^		
<150 cm	254	34.9
≥150 cm	474	65.1
Frequency of antenatal care		
No visit	17	2.3
1 to 3 visits	157	21.4
4 or more visits	558	76.2
Parity^a^		
Primiparous	314	42.8
Multiparous	420	57.2

^a^Missing data present

Table 2 lists the adverse obstetric symptoms as reported by our participants. In the antenatal period, 145 mothers (19.7 %) reported complications, compared to 204 (27.8 %) in the intranatal and 159 (21.6 %) in the postnatal period. During the antenatal period, foul smelling vaginal discharge (6.5 %), and swelling of hands (6.1 %) were the most common symptoms. During the intranatal period, prolonged labor (11.3 %), heavy bleeding (10.1 %) and swelling of hands (7.5 %) were commonly reported. During the postnatal period, swelling of hands (7.8 %) and high fever (4.2 %) were the main problems experienced by the mothers. No significant difference in the reported morbidities was found between primi-parous and multiparous woman.

**Table 2:** Adverse obstetric symptoms during antenatal, intranatal and postnatal periods

Problems	Frequency	Percentage
Antenatal	145	19.7
Some bleeding	63	8.5
Heavy bleeding	10	1.3
High fever	30	4.0
Foul smelling vaginal discharge	48	6.5
Swelling of hands	45	6.1
Fits or loss of consciousness	12	1.6
Intranatal	204	27.8
Heavy bleeding	74	10.1
Prolonged labor:12 to 23 hours	83	11.3
Prolonged labor: 24 to 47 hours	20	2.7
Prolonged labor: 48 hours or more	13	1.8
Retained placenta: 30 min to <1 hour	24	3.3
Retained placenta:1 hour or more	1	0.1
Swelling of hands	55	7.5
Fits or loss of consciousness	14	1.9
Postnatal	159	21.6
Heavy bleeding	12	1.6
High fever	31	4.2
Foul smelling discharge	26	3.5
Swelling of hands	57	7.8
Fits or loss of consciousness	12	1.6

A total of 81 (11.0 %) mothers reported a stillbirth previously, and 104 (14.1 %) mothers had cesarean section. There was a significant difference in cesarean delivery prevalence by location (*p* <0.001), with only 22 (5.8 %) of the 378 rural women had cesarean section compared to 82 (23.0 %) of the 357 urban women. On the other hand, prolonged labor (>12 hours) and heavy bleeding appeared to be more common in rural areas, where 72 (19.0 %) rural mothers reported having a prolonged labor as compared to 40 (11.2 %) urban mothers (*p* = 0.03). Similarly, 63 (16.7 %) rural mothers mentioned the signs of heavy bleeding during the intranatal period, as contrasted to only 11 (3.1 %) mothers residing in urban areas (*p* < 0.001). As shown in Table 3, logistic regression analysis confirmed the higher risk of incurring a cesarean delivery for these urban women relative to their rural counterparts (adjusted odds ratio 3.41, 95 % confidence interval 2.01 to 5.78).

**Table 3:** Factors associated with cesarean delivery in Western Nepal

Factors	Adjusted odds ratio^a^	*P*
Place of residence		<0.001
Rural	1.00	
Urban	3.41 (2.01, 5.78)	
Maternal education		<0.001
No education	1.00	
Primary to lower secondary	0.83 (0.38, 1.83)	
Secondary and above	2.56 (1.28, 5.14)	
Parity		0.010
Multiparous	1.00	
Primiparous	1.85 (1.16, 2.95)	

^a^Independent variables excluded from the stepwise logistic regression model were paternal occupation, wealth status, maternal age, frequency of antenatal care, and maternal height

## Discussion

An important finding is the unequal distribution of cesarean delivery across rural and urban areas. Rural mothers suffered more from adverse obstetric symptoms during the intranatal period. While the overall cesarean section rate of 14.1 % was within the World Health Organization’s recommended rate of 5 % to 15 % [19], a significantly higher rate of 23 % was evident in urban areas. Our result was consistent with previous studies conducted in South Asia [7, 20].

After a major reform in 2005, the Nepal health care system provides maternity services including cesarean delivery to its citizens free of charge at public facilities [21], and pays an allowance to cover travel expenses of the mother. With such initiatives, facility delivery rate has been successfully increased from 9 % in 2001 [22] to 35.3 % in 2011 [15], and especially high (73.1 %) in some accessible areas of Nepal [23]. Moreover, many public and private hospitals can now perform cesarean sections. Under these circumstances, the higher cesarean delivery rate in urban areas might merely reflect ‘on-demand’ or ‘provider initiated’ without a medical indication. Similar trends have been witnessed in South Asia and elsewhere [20]. Our findings on the rural–urban differentials in cesarean delivery rate and obstetric complications also reflect an uneven distribution of health services in Nepal. Provider-induced cesarean sections have potential adverse consequences, in terms of increasing the health care costs, and unnecessary occupation of hospital beds leading to overcrowding in tertiary hospitals. Further investigations would confirm whether those cesarean sections performed were indeed medically warranted.

Stillbirth is another problem for intervention that has received relatively little attention so far. Our observed prevalence of 11 % in Western Nepal was higher than the previous reported rate of 3.13 % in the Dhanusha district [24] but comparable with another hospital-based estimate of 13.3 % [25]. Recently, a verbal autopsy study to ascertain the causes of neonatal death in 2013 revealed that 37.9 % of the 551 deceased newborns were stillbirths, and fresh stillbirths accounted for 72.7 % of these cases [26]. Such in-depth assessment suggested the necessity to improve quality of care during childbirth, since 45 % of stillbirths could be averted by existing interventions [27]. This has important implications for Nepal because fresh stillbirths remain a major problem yet the quality of care is low during the intranatal period [28].

About one in five mothers reported adverse obstetric symptoms during the antenatal, intranatal and postnatal periods. Our result was similar to that of a previous study conducted in the Kaski district of central Nepal [7], but substantially lower than the 42.9 % postnatal morbidity incidence reported in Gadchiroli, India [29]. It should be remarked that the underlying settings were quite different, especially in terms of place of delivery. While the home delivery rate was only 11.8 % in the present study, it was 94.9 % in Gadchiroli, India. In light of the recent earthquakes, where more than 126,000 pregnant women were adversely affected or displaced, cesarean delivery services have not been restored in most of the earthquake affected districts, while two-third of birth centers were either destroyed or non-functional [30, 31], the progress made in maternal health since the 1990’s may be reversed so that urgent attention is required.

Heavy bleeding and prolonged labor are found to be common obstetric problems, both of which signal an immediate need for emergency obstetric care from skilled birth attendant and possible cesarean section. Although urban hospitals are capable to provide cesarean section and manage obstetric complications, such facilities and services are still lacking in the rural and remote mountainous areas of Nepal. Antenatal screening and early referral of rural mothers at risk would prevent maternal mortality and minimize the severe consequences of maternal morbidity. However, since some of the adverse maternal outcomes such as postpartum hemorrhage and sepsis cannot be predicted by screening alone, provision of emergency obstetric care within reach of the local community is important to save the lives of mothers. Since 2008/2009, the Ministry of Health and Population of Nepal has started allocating special funds for comprehensive emergency obstetric and neonatal care to cover the cost of equipment and health workforce for cesarean delivery [32]. It is still unknown whether this government initiative can reduce the burden of severe intranatal obstetric morbidity.

A major strength of the current study was the short recall time for the obstetric events since all interviews were conducted within 30 days postpartum. It also highlights the rural–urban difference in cesarean delivery rate and the prevalent obstetric complications in the Rupandehi district of Western Nepal, where maternal mortality was significantly higher than the national average [6]. Nevertheless, the self-reporting of signs and symptoms posed as the main limitation. In Nepal where the health information system is poorly maintained, information retrieved from hospital and government databases is unlikely to be accurate and reliable, even though record reviews have been cited as an important data source [33]. Therefore, self-reporting has been commonly relied upon in poor resource settings [7, 29, 34], despite the difficulty to obtain obstetric details from the mothers due to stigma, social and cultural reasons. In this study, the mothers were prompted with a comprehensive list of signs and symptoms that could occur during the antenatal, intranatal and postnatal periods, so that the reported prevalence of such events would reflect the true community situation. Another limitation concerns the lack of data on obstetric and neonatal outcomes such as stillbirth, pre-term birth and early neonatal deaths, without verbal autopsy, clinical audit and access to hospital records. Similarly, detailed investigation of the birth process, including treatment and medication used to augment labor, was not feasible given our budget constraint and available resources. Further studies are recommended to examine these issues especially stillbirths [35] and to confirm the impact of government incentives on cesarean rates.

## Conclusion

This study found that about one in five mothers had obstetric complications in Western Nepal. Furthermore, 11 % of mothers reported having a stillbirth, and 14 % of them underwent cesarean section. While prolonged labor and heavy bleeding were more common among rural mothers, the rate of cesarean section was more than four times higher in urban areas than in rural areas. Future public health interventions should focus on increasing access to obstetric services in rural areas, and the prevention of stillbirths through the provision of quality maternity care. Further investigations are also required to determine whether the cesarean sections conducted in urban areas are medically warranted or provider induced.

### Details of ethics approval

Nepal Health Research Council (773/2014) and the Human Research Ethics Committee of Curtin University (approval number HR184/2013).
